# Jordanian women’s experiences and constructions of labour and birth in different settings, over time and across generations: a qualitative study

**DOI:** 10.1186/s12884-020-03034-3

**Published:** 2020-06-10

**Authors:** Suha Abed Almajeed Abdallah Hussein, Hannah G. Dahlen, Olayide Ogunsiji, Virginia Schmied

**Affiliations:** School of Nursing and Midwifery, Western Sydney University, Locked Bag 1797, Penrith South, NSW 2751 Australia

**Keywords:** Labour pain, Pain relief, women's empowerment, Labour support, Human rights, Privacy

## Abstract

**Background:**

Overwhelmingly, women in Middle Eastern countries experience birth as dehumanising and disrespectful. Women’s stories can be a very powerful way of informing health services about the impact of the care they receive and can promote practice change. The aim of this study is to examine Jordanian women’s experiences and constructions of labour and birth in different settings (home, public and private hospitals in Jordan, and Australian public hospitals), over time and across generations.

**Method:**

A qualitative interpretive design was used. Data were collected by face-to-face semi-structured interviews with 27 Jordanian women. Of these women, 20 were living in Jordan (12 had given birth in the last five years and eight had birthed over 15 years ago) while seven were living in Australia (with birthing experience in both Jordan and Australia). Interview data were transcribed verbatim and analysed thematically.

**Results:**

Women’s birth experiences differed across settings and generations and were represented in the four themes: ‘Birth at home: a place of comfort and control’; ‘Public Hospital: you should not have to suffer’; ‘Private Hospital: buying control’ and ‘Australian maternity care: a mixed experience’. In each theme, the concepts: *Pain, Privacy, the Personal* and to a lesser extent, *Purity (cleanliness),* were present but experienced in different ways depending on the setting (home, public or private hospital) and the country.

**Conclusions:**

The findings demonstrate how meanings attributed to labour and birth, particularly the experience of pain, are produced in different settings, providing insights into the institutional management and social context of birth in Jordan and other Middle Eastern countries. In the public hospital environment in Jordan, women had no support and were treated disrespectfully. This was in stark contrast to women birthing at home only one generation before. Change is urgently needed to offer humanised birth in the Jordanian maternity system,

## Background

Overwhelmingly, women in Jordan and other Middle Eastern countries experience birth as dehumanising and disrespectful [1–4]. Studies report that there is little rapport between women and health professionals, that women lack information about the facilities where they will birth or the procedures that will be used, and women do not always give their consent for procedures [2, 3, 5, 6]. Typically, women labour in bed, alone with no access to a support person [2, 5] or privacy [3, 7], and receive limited support, or even abuse from health professionals [6].

This phenomenon of mistreatment by health professionals is not isolated to the Arab world [8–10]. Increasingly, there is evidence in high income countries (HIC) that women are being traumatised by their experience within maternity care systems, where intervention in birth is high and medicalisation is impinging on choice and humanised care [11–13]. We also know from studies in high income countries that the place of birth and the support women receive from their care providers profoundly impacts on women’s birth experience [14–16]. Studies in countries such as the United States, United Kingdom, and Australia are reporting that mistreatment during labour and birth is driving some women to seek alternatives and for some, this is to birth outside of the mainstream maternity system with no health professional present [13, 17, 18]. For some women, the hospital represents a riskier place to give birth than birthing at home [12].

The move to hospitalised birth took place in high income Western countries at the end of the 19th and early twentieth century, arguably for the safety of mother and infant [19]. Health professionals and women increasingly came to view hospitals as the safest place to give birth, particularly with access to technology and birth interventions [20, 21]. Although better birth outcomes have been the case for many women internationally, for others, medicalisation has created a disconnect between the pregnant woman and her body [20, 22]. This pattern appears to now be repeated in low and middle income countries including in the Middle East [22].

It was not until the late 70s and 80s that birth in Jordan and other Middle Eastern countries moved into hospital settings. Prior to this, giving birth at home was considered normal for Middle Eastern women [7, 23, 24]. Childbirth in Jordan was assisted by highly respected and experienced community women known as Dayas [24, 25]. In the 1970s, the Jordanian Ministry of Health acted to reduce maternal and infant mortality by, introducing a policy promoting hospital births, decreasing hospital costs to the women who birthed in a public hospital, and not allowing trained midwives to perform home births [24, 25]. At the same time, health care policies in Jordan discouraged home birth and traditional Dayas were only allowed to continue their work at home as a temporary process until they become too old to practise their profession [24]. Even as late as the 1990s, women still birthed at home, particularly women in rural areas.

The increasing evidence of mistreatment during labour and birth has garnered the attention of the World Health Organization (WHO) and Safer Motherhood [26]. The WHO [26] explicitly states that there is an urgent need for more evidence and action on ensuring that maternity care is respectful, maintains women’s dignity, and offers emotional support [26]. The WHO position statement on disrespectful care [27], Safe Motherhood For All [27], calls for maternity care that is comprehensive, participatory, rights-based, and uses evidence-based best practice. This statement is not just directed at low and middle income countries.

It is possible that the increasing dehumanisation of birth reported in Jordan, and other Middle Eastern countries, has coincided with the move from birth at home to birth in hospital facilities, and the increasing medicalisation of birth [1, 6]. Previous research by the authors [1], revealed that doctors in Jordan dominate maternity care practices in the hospital, direct policy related to maternity care, and midwives are directed to follow the obstetrician’s orders to manage normal births [1]. In this highly medical context, it was also evident that health professionals – doctors and midwives alike – appeared to view women disparagingly, believing that they lacked knowledge regarding the birth process [1].

Together with the WHO, authors suggest that significant change in maternity systems is needed to improve the care and the experience of women in Jordan and the Middle East [2, 3], though change will not be easy [1, 28]. As a first step, we argue it is important to understand the meanings that Jordanian women give to birth and to understanding their birthing experience, including how they view the birth environment, how they construct experiences of pain in labour and birth, and what influences these experiences. It is also important to examine what women themselves expect from maternity care and how they may want the system of care to change. To aid this understanding, it is important to explore women’s perceptions and experiences of how birth in Jordan has changed over time, including the impact of change in place of birth.

Therefore, this study aims to examine Jordanian women’s experiences and constructions of labour and birth in different settings (home, public and private hospitals in Jordan, and Australian public hospitals), over time and across generations. Women’s stories can be a very powerful way of informing health professionals and services about the impact of the care they receive and how this can be used to promote practice change. In this study, we draw on the theory of “Birth Territory” developed by Fahy and Parratt [15]. Birth Territory explicates the relationship between the birth environment or place of birth, and issues of power and control, and the way the woman experiences labour physiologically and emotionally. It may also inform service redesign.

## Methods

### Study design and theoretical underpinning

This was a qualitative interpretive study [29] and was informed by a feminist approach and the theory of Birth Territory articulated by Fahy and Parrott [15, 30]. Qualitative interpretive description was selected as the methodological approach to guide the design of the study and analysis of the data [31]. Interpretive description was developed by Thorne, Reimer, Kirkham and Mac-Donald Emes in 1997 [32] and is commonly used in health research to develop an understanding of, and the meanings that shape how individuals experience a health event such as an illness, or in this case, birth [33]. The interpretive descriptive methodological approach aligns philosophically with naturalistic inquiry as it recognises that the human experience is constructed by, and dependent on, the context in which a phenomenon is experienced, but also that there is the potential for shared realities [31]. The researcher understands that individual reality is a complex, subjective experience impacted by the context in which it is experienced and that they, the researcher, may influence the recollection of events being studied due to their interaction with participants [31, 33]. It is therefore an appropriate and broad approach to apply across cultures.

#### Birth territory

Fahy and Parratt [15] drew on Foucault’s work to theorise the birth room environment, describing the theory of ‘Birth Territory’. Birth Territory comprises two major concepts – ‘terrain’ and ‘jurisdiction’. Terrain denotes the physical features and geographical area of the birth space and comprises two sub-concepts, ‘sanctum’ and the ‘surveillance room’. The ‘sanctum’ is defined as a homely environment designed to optimise the privacy, ease, and comfort of the women; while the ‘surveillance room’ denotes a clinical environment that optimises, eases, and provides comfort for staff [15] p. 6. The concept of ‘jurisdiction’ refers to the power women have to do as they want within the birth environment. ‘Power’ is an energy that enables one to be able to do or obtain what one wants. Fahy and Parratt also identified ‘disciplinary power’ as a process that governs women’s behaviour and directs women to follow health professionals’ orders and be under their authority and control. Jurisdiction is comprised of four sub-concepts; ‘integrative power’, ‘disintegrative power’, ‘midwifery guardianship’, and ‘midwifery domination’. Integrative power refers to the power of all participants (women, midwives, and any other person) in the birth environment. It is a kind of power that focuses on the woman’s enhanced mind-body integration so that she can respond spontaneously to her bodily sensations during labour and birth. Women’s capacity to respond to her body is noticeably limited when they are not afforded privacy.

Fahy and Parratt [15] also described ‘midwifery guardianship’ as a form of ‘integrative power’ that involves midwives guarding the woman and her Birth Territory by controlling who accesses the birth space. This enables the woman to experience undisturbed labour and birth and promotes her sense of safety throughout, respecting her beliefs and attitudes during labour and birth. In Fahy and Parratt’s work, ‘disintegrative power’ was described as an ego-centred power that interferes with other forms of power within the birth environment. This power could be used by the woman, midwife, or any other person in the birth space. Regardless of who uses it, ‘disintegrative power’ limits women’s opportunity to feel, trust, and respond spontaneously to her bodily sensations.

Feminist concerns about patriarchy, hegemony and domination are fundamental to Fahy and Parratt’s work. Research informed by feminism prioritises women’s experiences in the context of their lives and focuses on addressing violations against women [34]. The aim of feminist research is to give women and their experiences a voice, and to transform lives based on those experiences [35, 36]. In this study, a feminist approach is used to inform data collection and to analyse and interpret the experiences and perspectives of Jordanian women during labour and birth.

### Study setting

This study was undertaken in Irbid, Jordan, and in Sydney, Australia. Jordan has a total population of 10,407,793 [37]. Irbid is the second largest city in Jordan, with a population of approximately two million. The number of births in Irbid in 2018 was 44.100 births (21.2% of all births in Jordan) [37]. In Jordan, almost all women (96%) receive maternity care in hospitals provided by midwives – this includes antenatal care in hospital clinics and community health centres [38]. Three out of four births are attended by a doctor and 82% of mothers receive post-natal care by a doctor, midwife, or nurse [39]. There are also increasing numbers of Jordanian women accessing services in private hospitals [40, 41].

Many Jordanian people have migrated to Australia and three-quarters of these reside in New South Wales [42]. Jordanian families primarily live in the west of Sydney and access services at nearby maternity units. Due to government policies in Australia, the rate of migration from Jordan is now much lower.

### Study participants and recruitment

To capture a diversity of Jordanian women’s voices, we sought the experiences of three groups of women. Recent mothers (RM), that is women who had become mothers in the past five years and experienced mothers (EM), being women who had given birth at least 15 years previously. In addition, to enhance understanding of the meaning of childbirth experiences for Jordanian women, we also sought women who had given birth to at least one child in Jordan and Australia. There was no restriction on how long ago women had birthed in Australia. These women are referred to as Australian Jordanian (experienced or recent mothers (AJEM or AJRM). This diversity of participants was selected to facilitate the exploration of the changes in perceptions of birth and birthing practices over generations and in different settings and countries.

To be included in the study, women had to have experienced a normal or instrumental birth. Women who had a caesarean section were excluded. This was because we were specifically interested in women’s experience, not only of labour, but of birth and the immediate postnatal period in different settings. Primiparous and multiparous women were both included in this study.

Ethical approval for the study was obtained from the University Human Research Ethics Committee (HREC) (Approval number H12048) in February 2017. Formal written approval was also obtained from the Jordanian Ministry of Health. Participants were recruited between March–May and September–October 2017 and December–February 2018.

Women were recruited through two major primary health care centres (Howara primary health care centre and Al Sareeh primary health care centre) in Irbid in Jordan. An information flyer about the study was placed on walls in both primary health care centres. The participant information sheet was distributed by administrative staff in the health care centres. This information was offered to both younger (RM) and older women (EM) who were mothers. The researcher (SH) was available to speak with interested women about the study in the waiting room of the health care centres and provided potential participants with the translated information sheet. The participants were reminded that participation was voluntary and those that provided written consent took part in the study. Some women, particularly the EM, were also recruited in the Jordanian community through word of mouth and women’s groups. In Sydney, information about the study was distributed through the Jordanian Women’s Association. Snowball sampling also occurred, with participants telling others about the study. Women who were interested in participating, were asked to contact the lead researcher either by email or phone to discuss the study. Written informed consent was obtained from all the participants.

### Data collection

Data were collected by face-to-face semi-structured interviews with the women. Open-ended questions and prompts were used to enable the participants to talk freely about their childbirth experience (see Table 1). Participants were asked to describe their birth experience(s) and explain their perceptions or the meanings they give to labour and birth. Their experience of pain associated with labour and birth was discussed, as well as how they managed the pain during labour and birth, including who was present or supported them. The interviews were arranged for a time that suited the women and was conducted in a mutually convenient place. The interviews lasted for 45 to 60 min and were digitally recorded. All the interviews were conducted in Arabic, appropriately, and respectfully by the first author. When interviews were conducted in a woman’s home or in a community setting, the first author took food and drink to share with the participants, which is an inherently Middle Eastern sign of respect and connection.

**Table 1:** Questions and key prompts used in the interviews

1. Can you describe your birth experiences for me? (prompt around where the participant gave birth; who was with her; what the services were like)?	
2. What does pain during labour and birth mean to you? How would you describe the pain and what did you do to manage this pain?	
3. Do you think that our attitudes to childbirth, including pain, are different from our mothers and why?	
4. (For RMs) How did your mother or mother in law influence how and where you birthed? What did they say to you about labour and birth during your pregnancy?	
5. (For EMs) How did your mother or mother in law influence how and where you birthed?	
6. Describe any particular cultural practices that are important to you concerning birth?	

### Data analysis

Interpretive description attempts to highlight the meanings and understanding of the research, using inductive analytic approaches, critical examination, and reflection, to highlight the characteristics and patterns in the research which may inform clinical applications of the results [31, 33]. In undertaking this analysis, we followed the steps of thematic analysis outlined by Braun, Clark, and Terry [43].

All data were transcribed verbatim and translated into English by a professional translator. Following this, back translation was conducted by the first author (SH) for validation purposes.

The transcriptions were read and re-read and the researchers engaged in a process of reflection to discover the meanings and experiences. Data were entered into the program, Quirkos, and coded [43]. Preliminary interpretations of the data were developed and participants’ words were used as appropriate to label initial codes. Emerging themes were identified and the linkages and relationships between the themes documented.

### Reflexivity

The first author (SH), is a Jordanian trained midwife and mother with a strong interest in improving women’s childbirth experiences in Middle Eastern countries. SH has experienced birth in different health settings in both Jordan and Australia. In most interviews the first author (SH) also shared her own birth experiences as appropriate with the participants. Using a feminist approach, it is important to share the experience of the researcher with the participants to encourage them to talk freely about their own experiences, but SH remained conscious to not dominate the conversation with her story or not take away from the story being told by the participant. As a member of the community, SH is embedded in the culture and shares the dominant language of the participant group. During data collection, SH had regular meetings via zoom with her co-researchers and supervision team to discuss the data collection and any challenges encountered in the field. These reflections were recorded in field notes. The co-researchers are not from the same cultural background and acted as a sounding board for SH, checking interpretations and explanations of the data and findings.

## Results

A total of 27 Jordanian women participated in the study, 12 participants were RM and eight were EM who were living in Jordan. In addition, seven Jordanian women living in Australia were interviewed. These seven women had given birth to at least one child in Jordan and one child in Australia. The RM and the EM were not related.

Recent mothers ages ranged between 18 and 37 years, (mean = 30 years). Experienced mothers ranged in age between 51 and 66 years (mean = 56 years). All the EM who lived in Jordan had given birth to at least one baby at home. In contrast, none of the EM who had given birth in Australia had given birth at home in Jordan or in Australia. None of the RM had experienced a home birth as this is now a rare event for Jordanian women. All of the EM, had also experienced birth in a public hospital with subsequent children, except one EM, and the contrasts or comparisons they offered during their interviews, have informed this analysis. In addition, four out of 11 EM had experienced birth in private hospitals. In contrast, 14 out of 16 RM had experienced birth in a public hospital and nine out of 16 RM had given birth in a private hospital in Jordan.

All of the EM and most of the RM were multiparous, just three of the RM were primiparous. The EM had between two and 10 children and the RM had between one and five children. Three out of 27 women had one child, four out of 27 women had two children, and four out of the 27 women had more than five children. Seven women had given birth in both Australia and Jordan. For some, their first birth was in Jordan, followed by a birth in Australia, and for others, their first birth occurred in Australia with a subsequent birth in Jordan. See Table 2 for characteristics of the participants.

**Table 2:** Characteristics of Participants

Age	
EM	Mean: 58 (Range: 51–66)
RM	Mean: 27 (Range: 18–37)
Number of children	
1	3
2	4
3	4
4	7
5	5
> 5	4
Number of children born at home	
EM	15
RM	0
Number of women birthed at home	
EM	8
RM	0
Number of women birthed in public hospital	
EM	10
RM	14
Number of women birthed in private hospital	
EM	4
RM	9

**Fig. 1:**
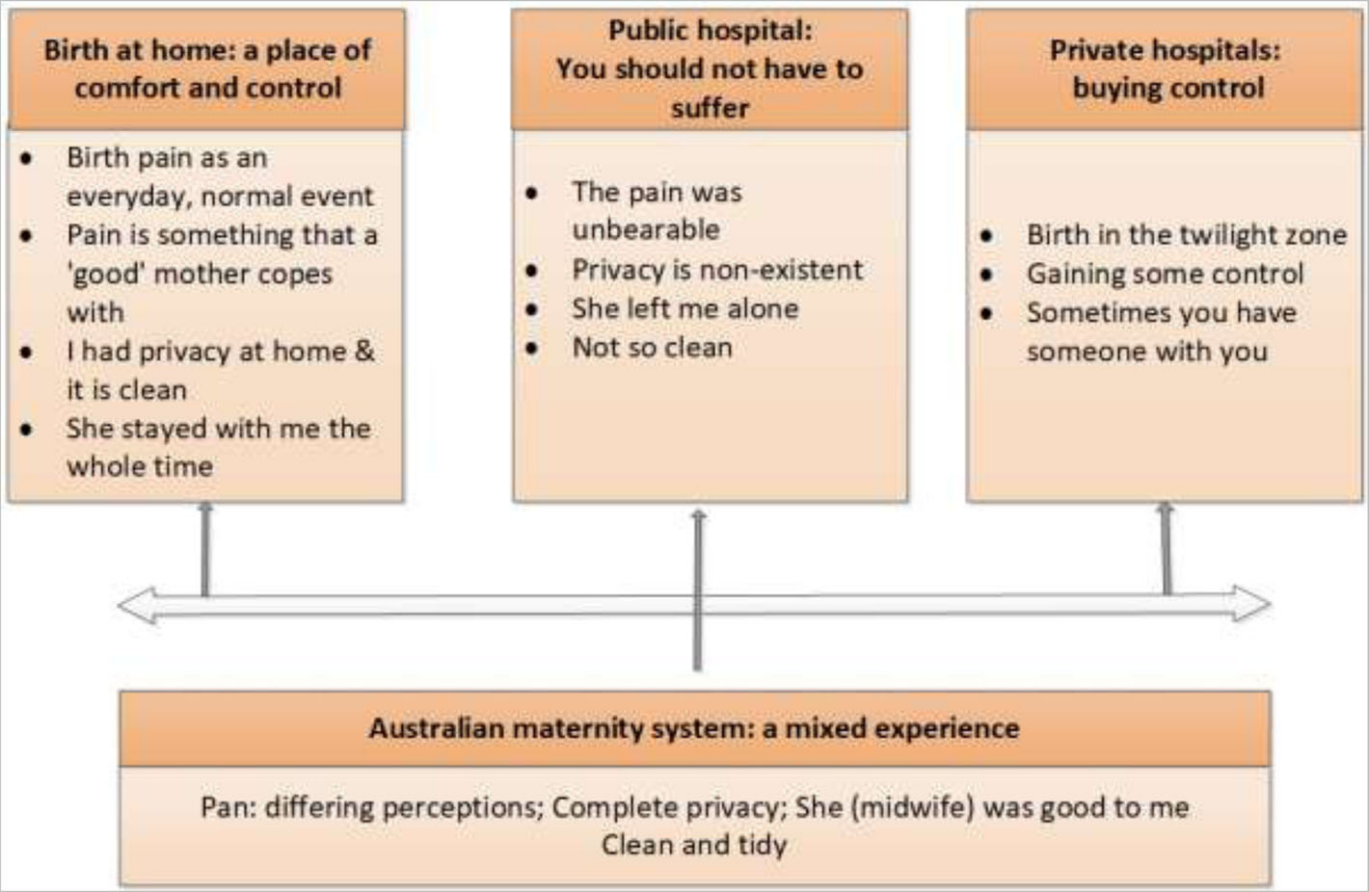
Study theme and sub-themes

The analysis revealed four major themes that reflected place of birth: ‘Birth at home: a place of comfort and control’; ‘Public hospital: you should not have to suffer’; ‘Private hospital: buying control’ and the fourth theme, ‘Australian maternity care: a mixed experience’, was a cross-cutting theme. There were also four common concepts across the women’s narratives - pain, privacy, the personal, and to a lesser extent, purity (cleanliness). These four concepts were present in women’s narratives over time, through generations and across cultures and in the different birthplaces – at home, in public hospitals and private hospitals in Jordan and in public hospitals in Australia. Each of these themes emphasises women’s desire to be in control over her labour and birth. Each of the themes is discussed separately, however, the experience of Jordanian women birthing in Australia is integrated across the first three themes as it provides a point of comparison to birth in Jordan. This is illustrated in Fig. 1.

### Birth at home. A place of comfort and control

Only the EM in this study had birthed at home, and they talked about home being a place of comfort, where they had the midwife and family around them, providing support. The women who gave birth at home viewed pain during labour as normal, something all women experience and they saw the way they tolerated it as representative of being a ‘good’ mother. Privacy and purity, or cleanliness, were also important and were consistently offered in the home environment.

#### Birth and labour pain is an everyday, normal event

For the EM, the dominant discourse around labour and birth and pain, was that birth was a normal, everyday event. It was seen as a part of life, occurring at home, where a woman recovers quickly and gets on with things*.* The following statements reflect this view: “*It is something women do, I am not the only woman that gives birth we all do. Everyone is the same”* (EM3) and *“It was the norm to go through that pain”* (EM 8). The following story from one EM suggests that women’s families also viewed birth as an everyday event:

*At the time I started having labour pain, my family in law were occupied as they were going to visit a girl and to offer a marriage proposal for my brother in law. I told them I had started labour and had pain, and I asked them to postpone it. But they called the midwife for me, and they left me alone in the home with the midwife and went to the girl’s house* (for the proposal) (EM 1).

The women who gave birth at home expected a speedy recovery: *“The next day after giving birth I was up and running, cooking and vacuuming”* (EM 5). For these mothers, home was an accepted place for birth, *“I would prefer* (to birth) *at home more than hospital because there is no need to go to the hospital”* (EM 4).

Most of the EM interviewed did not feel or remember the pain of labour and birth:

*I am the type. I do not feel a lot of pain. I was surprised at that time because I had a mild pain, not really pain and within half an hour I gave birth* (EM 1).

#### Pain is something that a ‘good’ mother copes with

Some of the EM believed it was important to handle the pain of childbirth to be a good mother. One mother stated:

*It is labour pain so no one can help you in relieving that pain, and I think to be a good mother you have to feel the pain of birth (EM 8)*.

One EM who gave birth to her last child in a private hospital, where pain medication seemed to be an assumed part of the service, appeared to regret having pain relief, as she also linked labour pain with being a good mother:

*In the private hospital they gave me some painkillers in the end, but I did not really need it and I think I can handle the pain and I should feel the pain to be a good mother* (EM 3).

The EM also talked about alternative, non-medicated approaches to pain relief, *“I had a hot shower and had some home hot herbs to increase my contractions and finish it quickly”* (EM 3), and *“The midwife helped me by doing perineal massage with olive oil; it helped me a lot”* (EM 7). Some of the strategies they used were spiritual in nature, *“This (the pain) scared me, but I prayed a lot”* (EM 5). One woman expressed her appreciation for the midwife who placed the Quran next to her in labour *“that made me relax”* (EM 4).

#### I had privacy at home and it is clean

Birth at home was also preferred by the EM because their privacy was protected and they did not have to share a room, as was often the case in the public, and sometimes the private hospitals. At home the midwife was viewed as guarding women’s privacy:

*She did not let anyone* (family members) *see me. She (midwife) asked them to stay in the same room but in a place where they could not see my body, to sit beside my head* (EM 7).

To offer greater privacy, some midwives preferred family members to wait outside the room, only allowing them permission for brief visits with the woman:

*The midwife at home gives me more privacy and care, I was in the room just with the midwife and my family members stayed in a room beside my room* (EM 3).

The EM also preferred giving birth in their own home as it was their domain and they knew it was clean, *“The home is cleaner* (than a public hospital)*, you are more comfortable in your own house and its environment”* (EM 1).

#### She stayed with me the whole time

While privacy was protected at home, women were not left alone. They either had support from the midwife or family; for example, participants described, *“She* (midwife) *stayed with me the whole time until I gave birth”* (EM 8) and another stated, *“Yes I had my mum and my mother in law”* (EM 6). When describing the people, they had around them at birth, only one EM mentioned her husband. She was clear that she liked the idea of having her family nearby, but not necessarily in the same room. At that time in Jordan, having men present at birth was considered unacceptable:

*My whole family is around me, my husband, and the other children, but they stayed in the other room. I just feel more comfortable that way* (EM 8).

The EM described the kindness and compassion of the midwives who were with them. *She* (the midwife) *was really good and very kind woman* (EM 4). One EM contrasted their experience with birth in a public hospital:

*She used to be patient with me, not like in the* (public) *hospital; they leave you alone in the room. Staying with one person the whole time during birth is better* (EM 2).

The women also stated that it was important to have practical support at home after the birth. EM 11 emphasised support was important because it could prevent postnatal depression,

*They help you, they make food and they stay around you most of the time and because of this help, you do not get depressed* (EM 1).

### Birth in public hospitals – you should not have to suffer!

Most of the RM and some of the EM interviewed had given birth in a public hospital in Jordan. The statement, *“It is what you do, you go to the hospital for the birth,”* represented the meaning that the RM gave to birthing in a hospital. It was also the place where RM expected to have their baby and where they expected to have a pain free birth, “*There is no need for the pain when you can take it away”* (RM 6). The RM did not believe that a woman had to suffer when giving birth:

*I did not want to experience the pain that’s why I went to the hospital to give birth… you should not have to suffer to be a mother* (RM 9).

However, this was not their experience, and in contrast to birth at home, women birthing in the public sector spoke vividly about the pain they experienced during labour and birth. In the context of a public hospital in Jordan, women’s needs for privacy, personal care and support and for purity or cleanliness were not met. In the public sector, women had no option to request what they wanted.

#### The pain was unbearable

Statements from the RM reflect their distress at the pain they experienced during labour and birth, “it *was unbearable”* (RM 4); *“It was a nightmare, and I really do not want to get pregnant anymore”* (RM 2)*.* The RM were particularly aggrieved that they were denied pain relief in the public hospital. Even if they asked the midwives for pain relief, the women stated they were not listened to, *“I asked for painkillers, they refused and told me ‘it is not good for me’”* (RM 6); and RM 11 stated, *“At least give the woman painkillers to help with the pain; they do not give it at all even if it is your first baby”*. Recent mother 2 described the response from midwives to her request for pain relief, *“Go to a private hospital if you need painkillers”.* The negative experiences of labour pain continued, particularly for first-time mothers, if they had an episiotomy during birth:

*It was very hard. Especially with my first baby because I had an episiotomy, it was very painful and hard to move around* (RM 7).

In contrast, the EM who also had at least one baby in a Jordanian public hospital continued to view labour pain as ‘normal’, and they tended to respond negatively to the idea of medicated pain relief, *“I would not have asked for painkillers as I see no need for it”* (EM 5). This was also the view of the A-JEM who had given birth in Australia. Even though in Australia, they had more options for medicated pain management, they maintained their opinion that pain was just part of labour and birth:

*I want to feel that feeling when the baby’s head comes out of my body. At that time, I felt that I was doing something extraordinary* (A-JEM 7).

Again, however, the perspective of RM birthing in Australia differed from the A-JEM, with the A-JRM appreciating the options for medicated pain relief:

*The midwife at the antenatal clinic explained to me what was available for pain relief in labour and I decided to choose the gas, I was happy about this* (A-JRM 5).

Yet, the use of medication was not necessarily a positive experience for those who accepted pain medication, *“I just do not like things that make me dizzy and that’s what these painkillers do”* (A-JRM 2).

Some of the RM birthing in Australia were also critical of midwives, noting that at times, the midwives refused to give them pain relief or had “tricked” the mothers into thinking they were receiving gas when the gas had not been turned on:

*My sister told the midwife to give me the gas, she (the midwife) said she does not need it. My sister told her just give it to her. She gave me the gas mask and we later noticed that she did not turn the gas on* (A-JRM 1).

The participants thought midwives sometimes withheld medicated pain relief, *“I am telling her I am in pain with no screaming but she refused. So, I pretty much gave birth on my own with no painkillers at all”* (A-JRM 4). Alternatively, A-JRM 2 believed that midwives used medication because they were busy, *“I felt the midwives gave the gas to me just so I keep busy with it and they can help others”.*

#### Privacy is non-existent

In the public hospital in Jordan there was no privacy. Experienced and recent mothers described the settings where they birthed in Jordan public hospitals, *“There are like six women in the same room, so no privacy”* (RM 6); and another woman stated, *“It was very easy for others to see me during labour and birth and that was very distressing for me”* (RM 10).

Some women did not voice concerns about privacy as it was what they expected. For example, one EM who had birthed in a public hospital considered sharing the same room with other women during birth as normal, but she insisted that the curtains should be drawn between the beds, *“No, everyone has to have their own room, but at least to have curtains closed all the time”* (EM 4).

The description ‘covered with a simple sheet’ represented a level of privacy that was acceptable to some women, *“They were very respectful and always covered me with the sheets”* (RM 7), while not for others: *“There was a sheet only covering the lower part of my body, but this is quite embarrassing, no privacy”* (RM 3).

This lack of privacy was intensified by the invasive approach of some health professionals during labour and birth. For example, women talked of being examined vaginally by many different people: “*It is embarrassing and makes you very uncomfortable”* (RM 4) and another stated:

*Yes, every time there was someone different examining me. That’s the problem. It was annoying and frustrating, and it hurts a lot* (RM 9).

The EM who birthed in public hospitals 20 years ago in Jordan, talked about being able to demand more privacy where no medical students were attending the birth:

*Only the midwife and the doctor were present in the labour room at that time, and this was a kind of privacy for me* (EM 1).

In contrast, the women who had birthed in a public hospital in Australia described the privacy they were afforded and they appreciated having their own room during labour and birth, *“I had a really big room, very clean and a bathroom for myself, it was very comfortable”* (A-JRM 5). They also appreciated the care that health staff took to maintain their privacy, *“Not one of my family is allowed to be in the labour room when the doctor is present for an internal checkup”* (A-JRM 5). These women also noted that mostly they were only cared for by one midwife and this, offered more privacy in contrast to Jordanian public hospitals, *“You have your own room and you have one midwife and she introduced herself to me”* (A-JRM 4).

#### She left me alone

The RM and EM who birthed in a public hospital in Jordan, reported that they were not allowed to have a support person with them during labour, *“They do not allow anyone to come in with me in the labour room”* (RM 7). Their request for support in public Jordanian hospitals was often met with the same response as when they asked for pain relief:

*When I told them to let my mum to come in with me in labour they said ‘go to private hospital if you want to let anyone to come in with you in labour’* (RM 9).

The women were critical of the Jordanian maternity service for not allowing them to have support people with them. One A-JEM was somewhat suspicious of this, and thought that the maternity staff in Jordan did not want to have outsiders interfering or observing what they do:

*They do not know how to treat the patient and the family. Maybe they do not want families to interfere in their business; that’s why they do not let people come in with their families* (A-JEM 3).

Women were asked who they would like to have with them during labour and birth. In contrast to the EM birthing at home, most RM wanted to have either their husband and or their mother with them:

*Probably my mum and my husband. That would be nice because they will help me and are there for me and support me* (RM 6).

When birthing in Australia, the A-JEM and A-JRM appreciated that the hospitals allowed support people to stay with them during labour and birth, *“It was lovely to have your family around you when you are in that much pain”* (A-JRM 4). However, women’s views on whether their husband should be present or not varied. One RM stated, *“I appreciated having my husband to stay with me the whole time in the labour room”* (A-JRM 2). Others preferred to have their mothers or female relatives instead of involving the husband for example, “*I do not mind my mum but my husband was not much help”* (A-J RM 4) and *“I do not recommend the husband to come in as he had a hard time dealing with what he saw.”* (A-J RM 5).

Only one of the RMs who had birthed in both Jordan and in Australia stated that she did not really want a support person at all in labour, *“I did not really feel I needed anyone as the midwives were good to me”* (A-JRM 1).

#### Not so clean

Not unexpectedly, it was also important to the participants that they birthed in a clean environment. Some of the mothers, both RM and EM, were shocked at how dirty the public hospital was in Jordan, *“The cleanliness in the hospitals is very poor, there were cockroaches on the floor moving between beds in the hospital”* (RM 11) and EM 8 stated, *“The bathroom was very dirty, it was so unclean, blood and dirt everywhere in the bathroom”.* While some RM considered cleanliness as sanitizing things in the hospital:

*One thing hospitals do not do is sanitise the cupboards, the beds that previous patients had been on. They should sanitize the beds, cupboards, and things around you* (RM 6).

One EM chose to give birth in a public hospital in Jordan rather than at home, because she thought it was cleaner, *“It was always clean but really it is the visitors and the women that dirtied the hospital, not that the hospital itself is dirty or unclean”* (EM 1).

All of the women who gave birth in Australia appreciated the cleanliness of the hospitals. As some of them stated that, *“Everything around is clean and tidy, so you do not need to worry”* (A-JRM 4), and,

*I can smell the cleaning products all around the hospital, so I can tell that this place is very clean and sanitized”* (A-JRM 1).

### Private hospitals - buying control

In the past 30 years there has been a dramatic increase in the number of births that occur in private hospitals in Jordan. In 2016, 58,019 babies were born in the private hospitals in Jordan. Thirteen participants in this study had given birth to at least one baby in a private hospital. This included nine RM and four EM. See table two for more information. All of the participants choosing a private hospital appeared to be seeking control over their birth experience. The prime reason that women gave for choosing to birth in a private hospital was for pain relief. Private hospitals were also described as cleaner, offering privacy through a private room, and with the possibility of having family with you.

#### Birth in the twilight zone

The RM who chose to birth in a private hospital reiterated their perspective that, *“You do not need to suffer as a mum”* and that they, *“Like(d) the idea of pain free birth”* and they preferred to pay to buy their comfort, privacy, and care in the private hospital:

*I preferred to pay money as long as I was in a comfortable place. So if I needed something they would give me* (RM 8).

In the Jordanian private hospitals, there are two options of pain relief in labour - epidural or ‘Dormicum and Valium’ which sedates women during labour and birth, much like ‘twilight sleep’ used in the past. Some RM liked the idea that they could be virtually asleep during the birth. Recent mother 8 stated, *“The injection. It calmed you and put you to sleep. That was right when the baby’s head was crowning”* and RM 12 also stated, *“I liked the painkillers I had it in the private hospital, I woke up after the baby was born”*. In the interviews, the women were asked how they felt when they not see their baby immediately at birth. This did not appear to worry the women. Recent mother 1 stated, *“No, it did not bother me at all, I just wanted to finish the pain”,* and another RM 4 said, *“I wanted that needle, my doctor gave it to me and I asked for that needle once I was admitted to the hospital”*.

In contrast however, the few EM who birthed in a private hospital did not favour being asleep during birth. As EM 3 stated, *“It disturbs the skin to skin bonding. I could not see my baby until three hours of giving birth”* and one EM 4 added:

*No I do not like the idea of being asleep when I gave birth. I did not see my daughter straight away and when I woke up I was confused and very tired to even hold my baby* (EM 4).

Four of the eight EM, had also birthed in a private hospital. In the interviews, these EM were asked about their decision to go to a private hospital. All four indicated this was because they could now not birth at home and they had not liked their experience in a public hospital, “*I was not going there (public hospital) again*” (EM 8). They also commented that the private hospital was cleaner, *“Big difference between the two* (private and public hospital), *with cleanliness”* (EM 4)*.* Following this birth, the EM questioned their decision, *“There was no need to go to a private hospital and pay a lot of money to only give birth there, it was just a few hours of labour pain”* (EM 3).

#### Gaining some control

Private hospitals offered privacy by not sharing the same room with other women, RM 5 stated, *“I had my own room at least, and I was in the same room the whole time, I was not moved to another room like public hospital”,* and RM 7 stated, *“They examined me vaginally every half an hour in the public hospitals but in the private hospitals it was only now and then”.*

Privacy however, was not always available in a private hospital. Two women living in Australia, who also had their first children in Australia, were surprised at the ‘practices in Jordan, even in a private hospital:

*I gave birth, there was no bed for me in the maternity ward, they put me in the corridor for two hours waiting for a bed in a private hospital, with people coming and going, and men also* (A-JEM 6).

Some of the RM and EM preferred giving birth in a private hospital in Jordan, not just for the privacy, but because they believed they choose a female doctor:

*If I was to get pregnant again I would go to a private hospital because I want a female doctor who would be with me the whole time* (RM 12).

The Jordanian women who birthed in Australia, also reported that they had the choice of a female or male doctor while giving birth in Australia, “*They do not let any male doctors inside the room without your permission”* (A-JRM 4) and A-JEM 3 stated:

*They ask you if you want a male doctor from your monthly visits during pregnancy. They see you wearing a scarf and they know you would prefer a female. So, they respect you and your religion*.

#### Sometimes you have someone with you

When birthing in a private hospital in Jordan, women had anticipated they would have someone with them. Most indicated that they were allowed one support person, *“Yes, they allowed my mum to come in with me the whole time during labour”* (RM 5), and *“The family members help and support me. It is good to have someone with you; it eases the pain on you”* (EM 4). Again, this was not always the case, and women reported that some private hospitals refused their request to have someone with them, *“No, they did not allow anyone to come in with me in labour”* (RM 8).

In the Jordanian private hospitals, women were also unlikely to have the same health professional during labour and birth, *“There were two or more midwives, often more. I had not the same midwife the whole time during the birth”* (RM 1).

## Discussion

This study aimed to examine Jordanian women’s experiences and constructions of labour and birth in different settings (home, public and private hospitals in Jordan, and Australian public hospitals), over time and across generations. The women who participated had given birth either at home, or in a public or private Jordanian hospital or an Australian hospital between 1979 and 2016. The key concepts that emerged from the analysis were; Pain, Privacy, the Personal, and to a lesser extent, Purity (cleanliness). Each concept was evident across the different generations of birthing women and in the different places (home and hospital) and countries they birthed in. Importantly, the experiences reported by participants demonstrate how meanings attributed to labour and birth, particularly the experience of pain, are produced and reproduced providing insights, not only into the medical and institutional management of birth but also the social context influencing decision-making around birth in Jordan and other Middle Eastern countries.

### Perception of birth and birth pain

Perceptions and experiences of labour and labour pain differed between the EM and RM. All of the EM believed that women’s bodies were designed for labour and birth and that medicated pain relief was not necessary. Feeling the pain of labour was associated with being a good mother. Alternatively, the RM argued that there is no reason why women should suffer pain in labour, that it was not the mark of a good mother and they were amenable to both technological and medicated approaches to birth. It appears in one generation we are seeing changes that occurred in many Western countries over two or even three generations of women.

Leap [44] suggests that women’s attitudes to labour pain can be divided into two models; ‘working with pain’ model and ‘pain relief’ model. The working with pain model clearly explains the EM’ perception and meaning of labour pain as a normal process, and that pain during labour plays an important physiological role in the production of the body’s natural pain-relieving opiates and endorphins. Internationally, studies indicate that women having home birth work differently with pain, and use a range of non-medicated approaches to pain, in contrast to women in hospital who report higher levels of pain, and have less options in terms of non-medicated pain relief [44, 45]. Experienced mothers in this study, and earlier research in the Middle East by Kabakian-Khasholian [46], show how childbirth was once viewed as a normal event in a woman’s life that usually happened at her own home, and was supported by the family members. Other researchers have also reported the non-medicated approaches to pain relief, such as having a hot shower, drinking warm herbs, or having a perineal massage with olive oil, is commonly used by Middle Eastern women [24, 47]. Experienced mothers also drew on their spiritual beliefs to support them through labour [23, 24, 47, 48]. For example, one EM described the midwife who read the Quran to her, reflecting the importance of spiritual beliefs in Jordanian women’s lives.

In contrast, Leap [44] argues that the dominant cultural approach to labour in high income countries is the ‘pain relief model’, where using some kind of medicated pain relief in labour is the norm [44, 49]. The ‘pain relief model’ clearly explains the RM’ perception of labour pain as unbearable, requiring medicated pain relief. The RM were adamant in their interviews that no woman should suffer the pain of labour, and that health professionals show kindness to women when they offer this pain relief. With these expectations, the RM birthing in the public hospitals in Jordan, and in some private hospitals, spoke with distress about their experience of pain during labour and birth. This experience is supported by the recent meta-synthesis of Middle Eastern women’s experiences of birth [6] that reported women in Middle Eastern countries experience birth as abusive, disrespectful and dehumanised. Women give birth without family present, but in crowded spaces, with no privacy and with limited options for medicated pain relief. There are also no options for alternative methods of working with pain, like immersion in water or other non-medicated methods. Instead women suffer. Some RM then turned to private hospitals to meet their needs, again they were disappointed. The meaning of labour pain for the EM and RM is subjective and dependent on what is available and this further supports the generational differences in the meaning and experience of labour pain.

#### Control & Power – birth territory

In Jordan and other Middle Eastern countries, the move from birth at home to hospital has disrupted factors such as one to one support and privacy known to assist the birth process and to support women to work with pain in labour [23, 24]. As introduced in this paper, the birth environment is a central concept in the theoretical work of midwifery researchers such as Fahy and Parratt [15] and Stenglin and Foureur [50]. In Birth Territory, as articulated by Fahy and Parratt’s [15], the midwife has a responsibility to ‘guard’ the birth territory for women – to ensure the environment is conducive to facilitating physiological birth. In this study, it was evident that at home the Daya was able to guard the women’s birth space, indeed some EM indicated that the Daya kept family members outside of the room. In this environment, the women were able to labour and birth as they wanted and they spoke positively about pain. Many studies report the benefit of continuity of care from midwives during labour and birth, describing how midwives support and guide women through pain, enabling them to feel confident and positive about their capabilities and inner strength [49, 51].

In contrast, in both the public and private hospitals in Jordan, and even in some instances in Australia, it was evident that women had no control over what happened to them during labour and birth. Fahy and Parratt [15] would explain, ‘disciplinary power’ in Jordanian hospitals governed women’s behaviour and directed women to follow midwives’ and doctors’ orders. In these birthing environments, women were not able to respond spontaneously to their bodily sensations during labour and birth. As described by Fahy and Parratt, this results in ‘disintegrative power’ which limits women’s opportunity to feel, trust and respond spontaneously to her bodily sensations [15], p. 6.

In this study, some women demonstrated their intention to seize control over their birthing experience by seeking care in a private hospital. In making this choice, the participants appeared to want to reclaim control over their birth experience, to control their labour pain, to ensure their privacy, and to assert the right to be accompanied by a trusted family member. In the private hospital environment, women reported being in control of some things such as privacy (own room) and pain where they can ask to be ‘knocked out’ using a form of twilight sleep [52]. They were also seen as cleaner and hence the desire for purity was also met more than in the public sector. Personal support needs, such as having a support person, was sometimes met but not always.

#### Seeking privacy

Research has demonstrated that a birth environment that provides women with privacy supports the hormonal processes of labour and birth [53, 54]. Numerous studies [7, 23, 55, 56], including one in Egypt [7], have reported that women feel more in control and emotionally secure birthing at home because they can maintain their privacy. This is consistent with the perception of the EM who had birthed at home and reported that they felt comfortable and secure in their own space at home. As described by the RM, Mohammad et al. [3] stated that in Jordan, most public hospitals are teaching hospitals, and labour wards are usually noisy and crowded with medical, nursing, and midwifery students. The situation is similar in Egypt [7] making the atmosphere very tense. Christiaens and Bracke [57] also indicated that participants voiced irritation at having to birth in a room with many other laboring women in public hospitals in Turkey.

#### Place of birth facilitates or disrupts personal support

The findings of this study revealed that at home women were not left alone. They either had support from the midwife or family. The lack of support from someone familiar and caring was a major concern for the women birthing in Jordanian public hospitals. It appeared that some women turned to a private hospital in Jordan, hoping they would have someone with them but were disappointed. In contrast, the Australian - Jordanian EM and RM appreciated that the hospitals in Australia allowed some people to stay with them during labour and birth. The importance of an environment with familiar and caring people, is also advocated clearly in the Birth Territory model [15]. There is significant evidence showing that support and continuity of care during labour decrease women’s need for pain relief and reduce the length of labour [3, 44, 58] including a Cochrane Systematic Review. This is also supported by studies in the Middle East [3, 58, 59]. A study conducted in Lebanon by Kabakian-Khasholian [60], showed that women greatly value the presence of someone they know and trust during labour. Similarly in the UAE, Mosallam et al. [58] observed a decreased length of labour and reduced need for pain relief and labour induction in women who had a supporter during labour and birth. In Jordan, women who had a female labour supporter were less likely to require pain relief and reported a satisfying childbirth experience compared with those who did not have a female supporter [61].

#### Purity and cleanliness

The EM in this study preferred giving birth in their own home as it was their domain and they knew it was clean. Some of the mothers, both recent and experienced, described public hospitals as being unclean. However, all of the recent and experienced mothers who gave birth in Australia appreciated the cleanliness of the hospitals in Australia. A good, physical birth environment influences women’s positive assessment of the childbirth services [62, 63]. Cleanliness and maintenance of hygiene were reported as determinants of satisfaction in studies in some LMIC [62, 64–67] and this includes good building infrastructure with water supply, beds, and cleanliness [64, 65]. In Bangladesh, one study reported that participants who rated the availability of a clean toilet as ‘good’ were significantly more satisfied with the care than those who rated these facilities as ‘poor’ [64].

### Implications for practice

There are immediate implications of this study for the training of midwifery and medical students as well as for professional development for trained staff. Staff and students require training to learn about women’s needs, the impact of birth trauma and to understand the effect that they have on women during one-to-one interactions. They also need to learn how the birth environment can affect birth outcomes and to understand how the health service shapes women’s experiences as well as the way in which they provide care. El-Nemer et al. [7] suggested that staff require education about compassionate care and Hatamleh et al. [2] stated that midwives need to learn how to advocate for women and facilitate supportive educational opportunities for women that value women’s knowledge and to build their knowledge and skills in normal birth.

Maternity services in Jordan also need to consider how the birth room environment might change. Research shows that birth environment, such as the layout of the rooms in hospitals, the sound levels, the light and cleanliness level and the presence of other people contribute both positively and negatively to the women’s experiences of labour and birth [68, 69].

Jordanian women may also benefit from educational opportunities, for example increasing the opportunity for prenatal education classes that are focused on women’s need with opportunities to explore options for non-medicated pain relief as well as discussing the purpose of pain in labour and birth [3, 4, 70, 71]. Oweis and Abushaikha [70] and Kridli et al. [71] both suggested that women should be offered childbirth education sessions that are culturally sensitive and present evidence-based information. This however implies that the onus is on women becoming more educated and agitating for change without any responsibility on health professionals and services to change.

Ultimately, change needs to occur at all levels of policy and practice, from the Ministry of Health, through to senior management in health services, maternity service leaders and those providing direct clinical care in birth units. Using the voice of women, such as those voices presented in this study, may be one way to shift attitudes. Change is urgently needed. The first author is now leading a participatory action research study bringing women together with health professionals to identify strategies for change. Further research is also needed to explore the impact of all these four concepts (pain, privacy, purity and the personal) on women’s birth narratives and stories.

### Strengths and limitations of the paper

This study offers an in-depth exploration of the concepts of pain, privacy, the personal (or social support) and purity or cleanliness relating to labour and birth reported by Jordanian women across different generations. It was unique in taking an intergenerational and cross country perspective which demonstrates how the birth environment impacts on women’s experiences. The study offered an opportunity to explore the hegemonic nature of women’s birthing experience in Jordan.

This study has several limitations that should be noted. First, the study was conducted in Irbid, Jordan where the EM and RM either birthed in the same public or private hospital and therefore the findings may not be generalisable to other women in Jordan and the Middle East. While the invitation to participate in the study was open to all, only 27 Jordanian women agreed to be interviewed in this study. This is a small number of women from one country and may not represent the views of other women. In addition, while we had set the criteria for RM to have had their last child in the past 5 years, we found some women were very keen to participate who had their last child between six to 8 years previously. We agreed to include these women in the study. However, gaining insight from 27 participants provides a platform to understand Jordanian women’s experience of labour and birth pain. Another limitation was that the participating women self-selected, so they agreed to be interviewed and women who did not agree may have had different stories to tell. Finally, women may not necessarily have felt comfortable discussing everything they had experienced.

## Conclusion

This study aimed to examine Jordanian women’s experiences and constructions of labour and birth in different settings (home, public and private hospitals in Jordan, and Australian public hospitals), over time and across generations. The theory of “Birth Territory” was used to explicate the relationship between the birth environment, issues of power and control and women’s birth experiences and use of medicated pain relief. This study showed how women’s perceptions and experiences of birth in Jordan have changed rapidly across one to two generations of women. Despite the benefits of more highly trained maternity staff and access to technology, it appears that in general, Jordanian women are unhappy with the way they are treated in birth and many report traumatic experiences. This study exposes the high level of disregard for women and their need for pain relief, privacy, and support during birth in public and private hospitals in Jordan. This disregard is deeply embedded in a maternity system dominated by medicine and associated patriarchal cultural practices and beliefs.

Action is needed at all levels from policy, education and practice, however, in this context, making change will not easy. In the short-term, training about compassionate care and human rights in childbirth should be provided to midwifery and medical students at university and professional development for maternity staff. Hearing Jordanian women’s stories may be a very powerful way of informing health professionals and services about the impact of the care they receive and how this can be used to promote practice change. In the longer-term, it is likely that women themselves will need to demand the change.

## Data Availability

The datasets used and/or analysed during the current study are available from the corresponding author on reasonable request.

## Abbreviations

AJM: Australian Jordanian Mothers
A-JEM: Australian-Jordanian Experienced Mothers
A-JRM: Australian-Jordanian Recent Mothers
EM: Experienced Mothers
HIC: High Income Countries
HREC: Human Research Ethics & Committee
LMIC: Low and Middle Income Countries
RM: Recent Mothers
WHO: World Health Organisation
